# Supplementary material for: Refinement in the European Union: A Systematic Review
Source: Animals (Basel). 2022 Nov 23;12(23):3263. doi: 10.3390/ani12233263 (PMC9735736; doi:10.3390/ani12233263)
Supplement: Supplementary file 1 [file animals-12-03263-s001.zip › Supplementary Table 2.pdf]

animals

MDPI

Country	Financial support				
	No-funding	EU	National	Institutional	Private Foundation
Austria	3				
Belgium	1	1			
Czech Republic		1	1		
Denmark	1	1	1		1
France		1	2	1	
Germany	5	1	7	4	1
Hungary		1			
Italy		1	1		
Norway		1			
Spain	3				
Sweden	2	1			1
United Kingdom	3	4	16	2	
